# Elevated HDAC activity and altered histone phospho-acetylation confer acquired radio-resistant phenotype to breast cancer cells

**DOI:** 10.1186/s13148-019-0800-4

**Published:** 2020-01-03

**Authors:** Asmita Sharda, Mudasir Rashid, Sanket Girish Shah, Ajit Kumar Sharma, Saurav Raj Singh, Poonam Gera, Murali Krishna Chilkapati, Sanjay Gupta

**Affiliations:** 1 Epigenetics and Chromatin Biology Group, Gupta Lab, Cancer Research Institute, Advanced Centre for Treatment, Research and Education in Cancer (ACTREC), Tata Memorial Centre (TMC), Kharghar, Navi Mumbai, MH 410210 India; 2 Homi Bhabha National Institute, Training School Complex, Anushakti Nagar, Mumbai, MH 400085 India; 3 Department of Oncology, Faculty of Medicine and Dentistry, University of Alberta, 11560 University Avenue, Edmonton, Alberta T6G 1Z2 Canada; 4 Chilkapati Laboratory, Advanced Centre for Treatment, Research and Education in Cancer (ACTREC), Tata Memorial Centre (TMC), Kharghar, Navi Mumbai, MH 410210 India; 5 Biorepository, Advanced Centre for Treatment, Research and Education in Cancer (ACTREC), Tata Memorial Centre (TMC), Kharghar, Navi Mumbai, MH 410210 India

**Keywords:** Breast cancer, Chromatin, Histone deacetylase, Histone post-translational modifications, Radio-resistance, Radiotherapy, Valproic acid

## Abstract

**Background:**

Poor-responsiveness of tumors to radiotherapy is a major clinical problem. Owing to the dynamic nature of the epigenome, the identification and targeting of potential epigenetic modifiers may be helpful to curb radio-resistance. This requires a detailed exploration of the epigenetic changes that occur during the acquirement of radio-resistance. Such an understanding can be applied for effective utilization of treatment adjuncts to enhance the efficacy of radiotherapy and reduce the incidence of tumor recurrence.

**Results:**

This study explored the epigenetic alterations that occur during the acquirement of radio-resistance. Sequential irradiation of MCF7 breast cancer cell line up to 20 Gy generated a radio-resistant model. Micrococcal nuclease digestion demonstrated the presence of compact chromatin architecture coupled with decreased levels of histone PTMs H3K9ac, H3K27 ac, and H3S10pK14ac in the G_0_/G_1_ and mitotic cell cycle phases of the radio-resistant cells. Further investigation revealed that the radio-resistant population possessed high HDAC and low HAT activity, thus making them suitable candidates for HDAC inhibitor–based radio-sensitization. Treatment of radio-resistant cells with HDAC inhibitor valproic acid led to the retention of γH2AX and decreased H3S10p after irradiation. Additionally, an analysis of 38 human patient samples obtained from 8 different tumor types showed variable tumor HDAC activity, thus demonstrating inter-tumoral epigenetic heterogeneity in a patient population.

**Conclusion:**

The study revealed that an imbalance of HAT and HDAC activities led to the loss of site-specific histone acetylation and chromatin compaction as breast cancer cells acquired radio-resistance. Due to variation in the tumor HDAC activity among patients, our report suggests performing a prior assessment of the tumor epigenome to maximize the benefit of HDAC inhibitor–based radio-sensitization.

**Graphical abstract:**

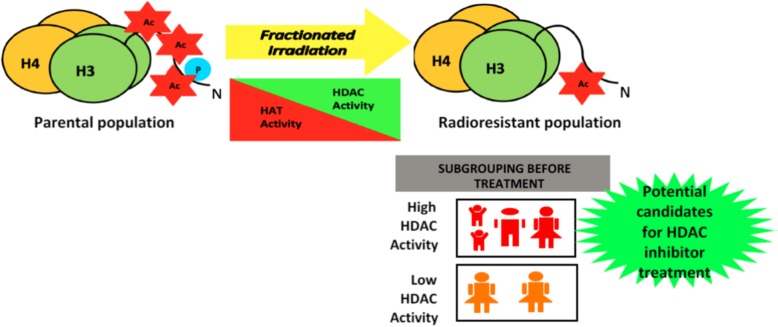

## Background

Breast cancer is the most common cancer among women in India and worldwide, with an annual increase in both incidence and mortality rate [1, 2]. Surgery and radiotherapy are the mainstay for loco-regional control, along with an additional chemo/hormone therapy to tackle possible distant metastasis [3]. Radiotherapy post mastectomy has been associated with an increased overall survival, decreased mortality and recurrence rate of lymph node–positive breast cancer patients [4]. The major aspects that govern tumor response to radiation (also called the 4R’s of radiobiology) are repair, redistribution, repopulation, and reoxygenation. Yet what still intrigues clinicians is tumor recurrence or poor treatment response to radiotherapy. This elusive phenomenon, called radio-resistance, has been termed as the 5th R of radiobiology [5].

Attempts to elucidate the genetic, proteomic and transcriptomic determinants of radio-resistance revealed altered gene expression patterns and protein–protein interaction networks [6–9]. However, these studies have a major caveat of not taking the cell cycle phase–specific alterations in gene expression and protein profile into consideration. A radio-sensitivity signature, comprising of DNA repair and cell cycle–related genes (identified in breast cancer cell lines and validated in an independent patient dataset) was successful in predicting local recurrence [10]. Another study reported an increase in expression of genes involved in epithelial-to-mesenchymal (EMT) transition, angiogenesis, and proliferation in a highly radio-resistant tumor, glioblastoma [11]. Also, a study of genomic alterations in breast cancer patients revealed an association of phosphatidylinositol-4, 5-bisphosphate 3-kinase catalytic subunit alpha (PIK3CA) mutation with loco-regional recurrence in case of breast cancer [12]. Thus, identification of specific radio-resistance-associated signature patterns can be of immense utility to develop novel treatment strategies or introduce “customized” radio-sensitizers to prevent recurrence [13].

Major biological factors like the tumor microenvironment [14], cell cycle phase [15] and DNA repair pathways [16, 17] strongly govern the outcome of radiotherapy. Carefully coordinated epigenetic events that lead to changed histone post-translational modification (PTMs) levels are crucial during the initiation, repair as well as the termination stages of the DNA damage response (DDR) [18–20]. Alterations of histone PTMs during DDR could possibly affect the cellular response to radiation, thereby influencing the acquirement of radio-resistance. Indeed, studies on human skin fibroblasts, 3D cultures of A549 human lung carcinoma cell line, and salt-based solid-phase chromatin manipulation have shown an increase in heterochromatin content to be associated with radio-resistance [21–23]. Human lung carcinoma cells exposed to linear energy transfer (LET) radiation displayed an overall increase in heterochromatinization and higher levels of H3K9me3 [24]. Loss of H4K20me3 was also observed in the thymus of mice exposed to low-dose total body irradiation [25]. Additionally, our group has previously demonstrated the association of H3S10p dephosphorylation and deacetylation of residues H3K9ac, H3K14ac, and H3K56ac during radiation-induced DNA damage [26]. This evidence strongly points towards the possibility of a distinct epigenetic signature that develops during radiotherapy. Epigenome-modifying enzymes that influence gene expression (by altering histone PTM levels) may also regulate resistance towards radiation. Small molecule inhibitors like valproic acid (VPA), trichostatin A, and suberoylanilide hydroxamic acid against histone deacetylases (HDACs) have sparked considerable interest due to their potent radio-sensitization ability [27–31]. Thus, an understanding of the epigenetic alterations during radiotherapy may be useful in combating radio-resistance.

In light of the available reports, in this study, radio-resistant MCF7 and MDA-MB231 breast cancer sub-cell lines were developed to investigate the epigenetic alterations that occur during radiotherapy. Changes in the epigenome pointed towards a more condensed chromatin architecture, significant alterations in histone H3 acetylation and phosphorylation as well as histone acetyl transferase (HAT) and HDAC activities. These radio-resistant cells were effective targets for sensitization using HDAC inhibitor VPA. We also provide evidence of variable HDAC activity in human tumor samples. Finally, we propose the necessity of patient categorization (based on epigenetic background) to maximize the effectiveness of epi-drug therapy and reduce the rate of tumor recurrence.

## Results

### Radio-resistant cells display alterations of various physiological processes

**Fig. 1:**
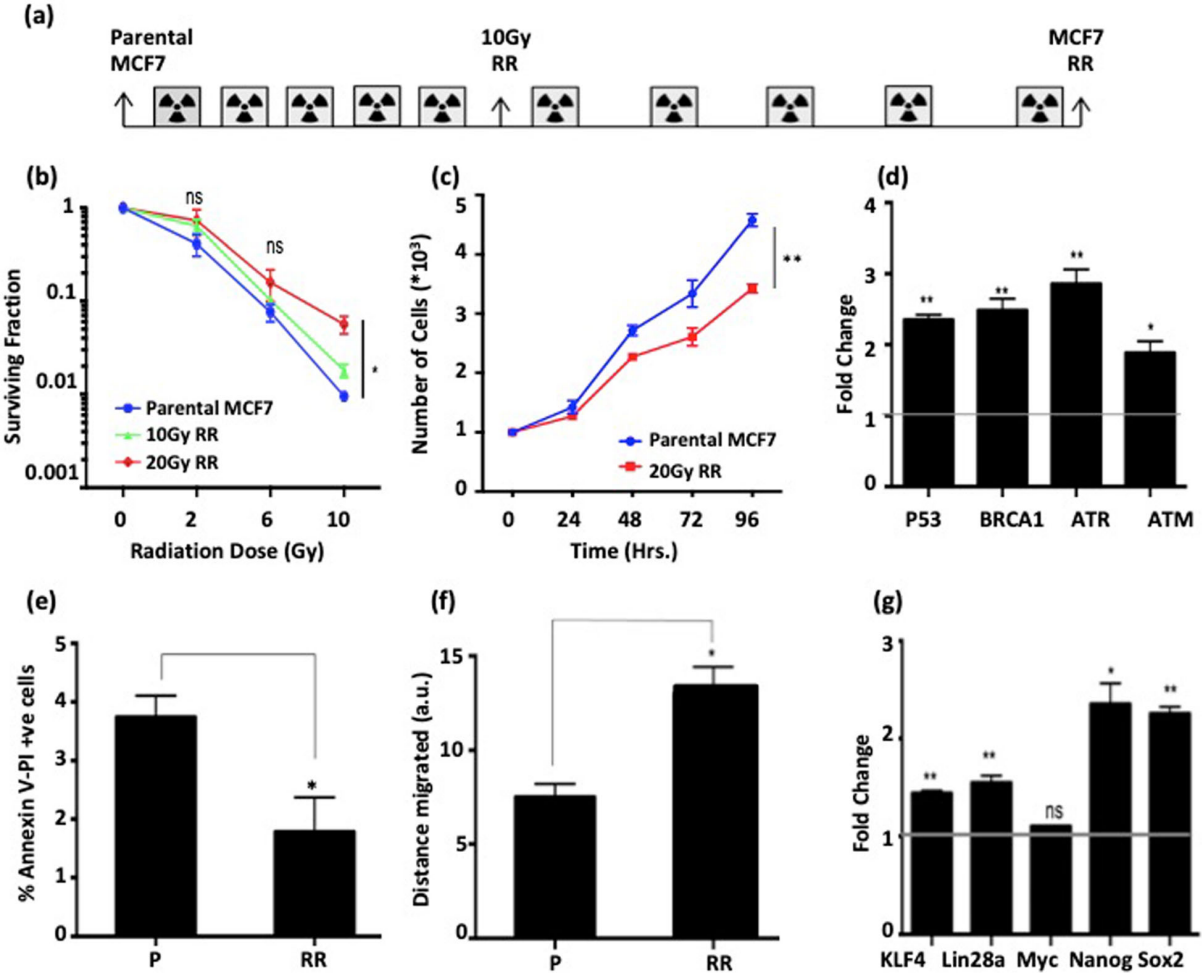
Radio-resistant cells display alterations of various physiological processes. **a** Schematic representation of generation of MCF7-RR. **b** Graph depicting surviving fraction of P and RR at different radiation doses. **c** Graphical representation of proliferation rate of P and RR. **d** Real-time PCR based expression analysis of DNA repair–related genes in P and RR. Levels normalized to MCF7-parental. Fold change 1 depicts levels of parental MCF7. **e** Graph depicting percentage of AnnexinV and Propidium Iodide (PI) dual positive cells in P and RR. **f** Quantitative representation of cell migration potential of P and RR, plotted as distance migrated after 20 h. **g** Real-time PCR–based expression analysis of stemness-related genes in P and RR. Levels normalized to MCF7-parental. Fold change 1 depicts levels of parental MCF7. Parental MCF7 is denoted as “P” and radioresistant cell line is denoted as “RR”. Statistical analysis is done by Student’s *t* test. *N* = 3 for all experiments. ^*^p < 0.05, ^**^p < 0.01, n.s.—not significant, a.u.—arbitrary units, Gy—Gray and Hrs.—Hours. Error bars represent ± S.D. of 3 experiments

MCF7 breast cancer cell line was subjected to radiation (10 rounds of 2 Gy each) and a radio-resistant sub-cell line (designated as MCF7-RR) was generated (Fig. 1a). Similarly, another radio-resistant sub-cell line of MDA-MB231 breast cancer cell line (designated as 231RR) was generated. Clonogenic assay revealed an enhanced dose-dependent survival for both MCF7-RR (Fig. 1b, Additional file 1a and b) and 231RR (Additional file 3a and b). MCF7-RR cells demonstrated a decrease in their proliferation capacity compared with parental cells in a time-dependent manner (Fig. 1c). Also, an increase in the expression of DNA repair-related genes was observed in MCF7-RR, which might contribute to enhanced radio-resistance potential of these cells (Fig. 1d). A comparative analysis revealed decreased levels of AnnexinV-PI-positive population (depicting late apoptotic cells) in MCF7-RR (1.36%) compared with parental MCF7 (3.48%) (Fig. 1e and Additional file 1c). Further, an assessment of cell migration potential revealed an enhanced migration capacity of MCF7-RR (Fig. 1f and Additional file 1d). This corroborated a previous study that reported an epithelial-to-mesenchymal transition (EMT) to be induced in breast cancer cells as an effect of radiation [32]. MCF7-RR also showed an increase in expression of transcription factors KLF4, SOX2, and Lin28A gene, which might be responsible for conferring enhanced migration capacity of MCF7-RR (Fig. 1g).

### Radio-resistant cells possess distinct morphological and Raman spectral features

**Fig. 2:**
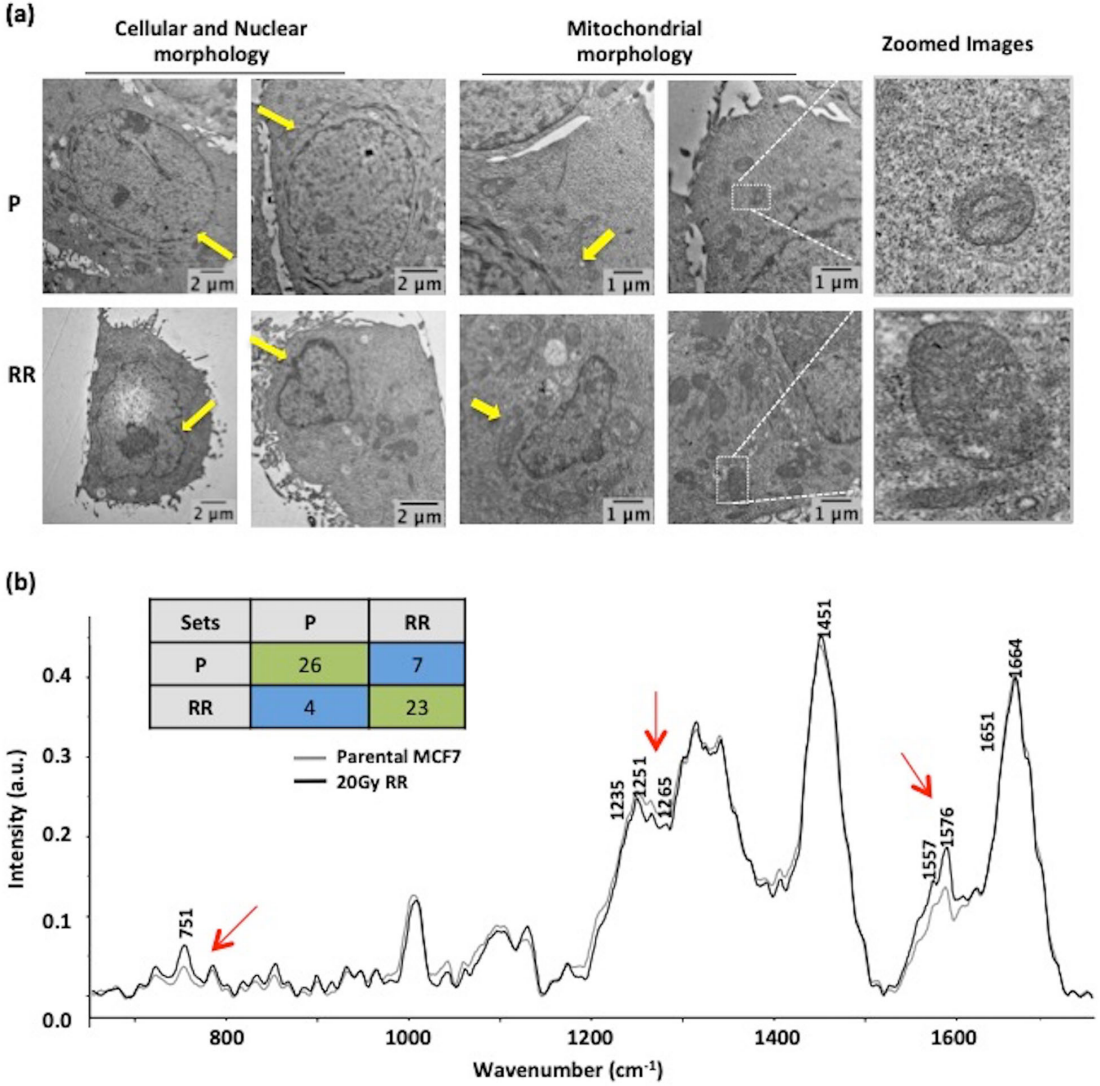
Radio-resistant cells possess distinct morphological and Raman spectral features. **a** Transmission electron microscopy images of P and RR show nuclear (left panel) and mitochondrial morphology (right panel and zoomed images). Yellow arrows point towards observed changes. **b** Raman spectra (650–1750 cm^− 1^) of P (gray) and RR (black). Altered spectral features are highlighted at 751, 1265 and 1576 cm^− 1^ by red arrows. A total of 30 spectra analyzed for P and RR are depicted in the confusion matrix as a box indicating spectral matching between P and RR. Boxes in green depict matched spectra and blue depicts unmatched spectra. Parental MCF7 is denoted as “P” and radioresistant cell line is denoted as “RR” and a.u. is arbitrary units. Images were taken at magnification × 1000 and scale bar depicts 2 μm for cellular and nuclear morphology and 1 μm for mitochondrial morphology, respectively

Confocal and electron microscopy analysis of MCF7-RR revealed alterations in cellular morphology and nuclear architecture. The radio-resistant population displayed aberrations in overall nuclear morphology, as opposed to the well-rounded geometry of parental MCF7 nucleus (depicted by yellow arrows in Fig. 2a). A reduction in the nuclear size was also observed in MCF7-RR (Fig. 2a, Additional file 2a {DAPI channel} and c). Staining with PKH lipophilic dye revealed the loss of a well-defined cellular shape in the case of MCF7-RR, compared with parental cells (Additional file 2b) An aberration in organization and the intra-cellular arrangement of cytoskeletal protein α-tubulin was also evident (Additional file 2a). These observations indicated a distorted cellular and nuclear morphology upon fractionated radiation exposure. Previous reports provide evidence about altered mitochondrial function and abundance post radiation exposure [33–35]. MCF7-RR also showed an increase in the mitochondrial size (represented by yellow arrows in Fig. 2a and zoomed images). These observations indicated effects of ionizing radiation on nuclear morphology and subcellular organelles. In addition to distinguishing morphological features, MCF7-RR also possessed biochemical alterations. As analyzed by Raman spectroscopy, MCF7-RR showed changes in the amide I region (~ 1650–1665 cm^− 1^), amide III region (~ 1235–1265 cm^− 1^) and δCH2 deformation (around 1450 cm^− 1^) of the Raman spectra (Fig. 2b as marked by red arrows). The amino acid Tryptophan (751 cm^− 1^ and 1557 cm^− 1^) also displayed a subtle increase in MCF7-RR. The confusion matrix (presented as a box in Fig. 2b) demonstrated 26/30 (66.67%) and 23/30 (76.67%) correct spectral classification for parental and radio-resistant MCF7, respectively. These observations hint towards development of distinct morphological and biochemical features in response to radiation exposure. Such an analysis might aid in microscopy- and spectroscopy-based identification of a radio-resistant population.

### Radio-resistant cells possess compact chromatin architecture

**Fig. 3:**
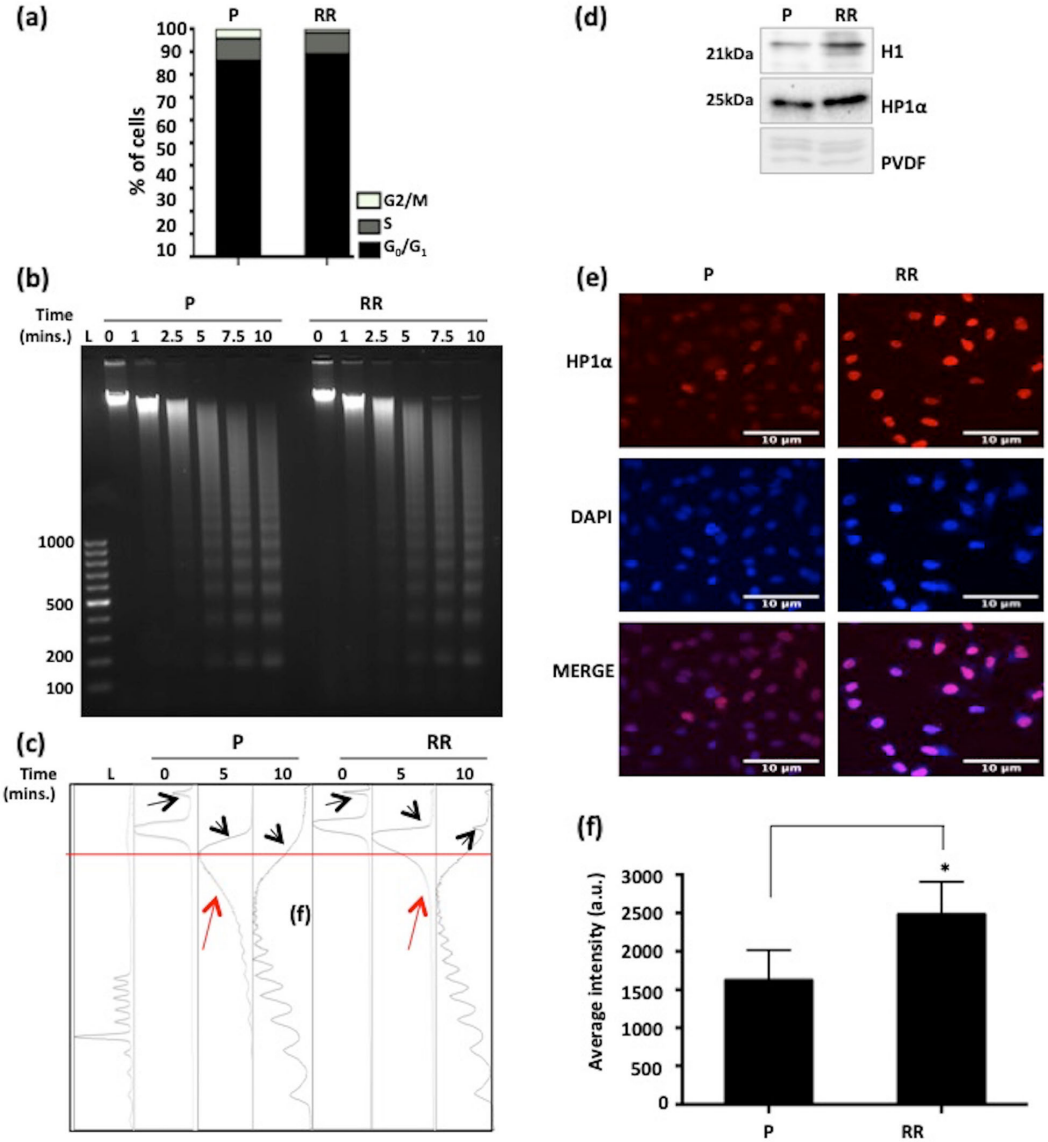
Change in global chromatin architecture of radio-resistant cells. **a** Cell cycle profile of P and RR synchronized in G_0_/G_1_ phase. **b** Chromatin architecture alterations analyzed by micrococcal nuclease (MNase) assay resolved on 1.8% TAE-agarose gel to visualize the digestion pattern. Time points indicate the duration of incubation of nuclei with MNase. **c** Densitometry based representation of MNase digestion. Black arrows point to areas of overall change in chromatin architecture between P and RR. Red horizontal line and red arrows point towards conversion of high molecular weight genomic DNA to polynucleosomes at 5-min time point. **d** Western blots depict levels of histone H1 and HP1α in P and RR. PVDF membrane showing core histones serves as loading control. **e** Representative z-stack projection images of HP1α immunofluorescence in P and RR. DAPI staining depicts nuclei. Scale bar - 10 μm (**f**) Graph represents the average nuclear intensity of HP1α quantified from *n* = 50 nuclei from different fields. Scale bar- 10 μm. Parental MCF7 is denoted as “P” and radioresistant cell line is denoted as “RR.” Statistical analysis is done by Student’s *t* test. L is 100 bp ladder. ^*^p < 0.05, ^**^p < 0.01, a.u.—arbitrary units and mins.—minutes. Images were processed using LSM browser software and Image J for confocal microscopy and MNase densitometry, respectively

The chromatin organization of MCF7-RR and 231RR was analyzed by Micrococcal nuclease (MNase) digestion. The altered MNase digestion pattern of MCF7-RR and 231RR could be attributed to prolonged radiation exposure, and may not be due to any difference in the cell cycle profile of parental and radio-resistant cells (Fig. 3a and Additional file 3e). The data revealed no significant difference in the time of appearance or intensity of mono- and di-nucleosomes in MCF7-RR, compared with parental cells (Fig. 3b and c). Also, the average mononucleosome length (~ 164 bp) remained same and consistent with the time of digestion. However, the digestion pattern suggested that conversion of genomic DNA to polynucleosomes was faster in parental MCF7 compared with MCF7-RR (indicated by red arrows in Fig. 3c). Also, there was a presence of high molecular weight MNase-resistant undigested DNA (indicated by black arrows in Fig. 3c). In 231RR cells, though the rate of formation of mononucleosomes was faster than parental cells, and this population also showed the presence of a high molecular weight MNase-resistant undigested DNA as observed in MCF7-RR (Additional file 3c and d, changes pointed by red arrows). Additionally, MCF7-RR showed an increased intensity and more uniform distribution of HP1α (Heterochromatin Protein 1 α) throughout the nucleus, compared with MCF7 parental cells (Fig. 3d–f). This indicates chromatin compaction in radio-resistant breast cancer cells and enhanced heterochromatinization during the acquirement of radio-resistance.

### Radio-resistant population exhibit global histone hypo-acetylation, downregulation of MAPK pathway and altered activities of HDACs and HATs

**Fig. 4:**
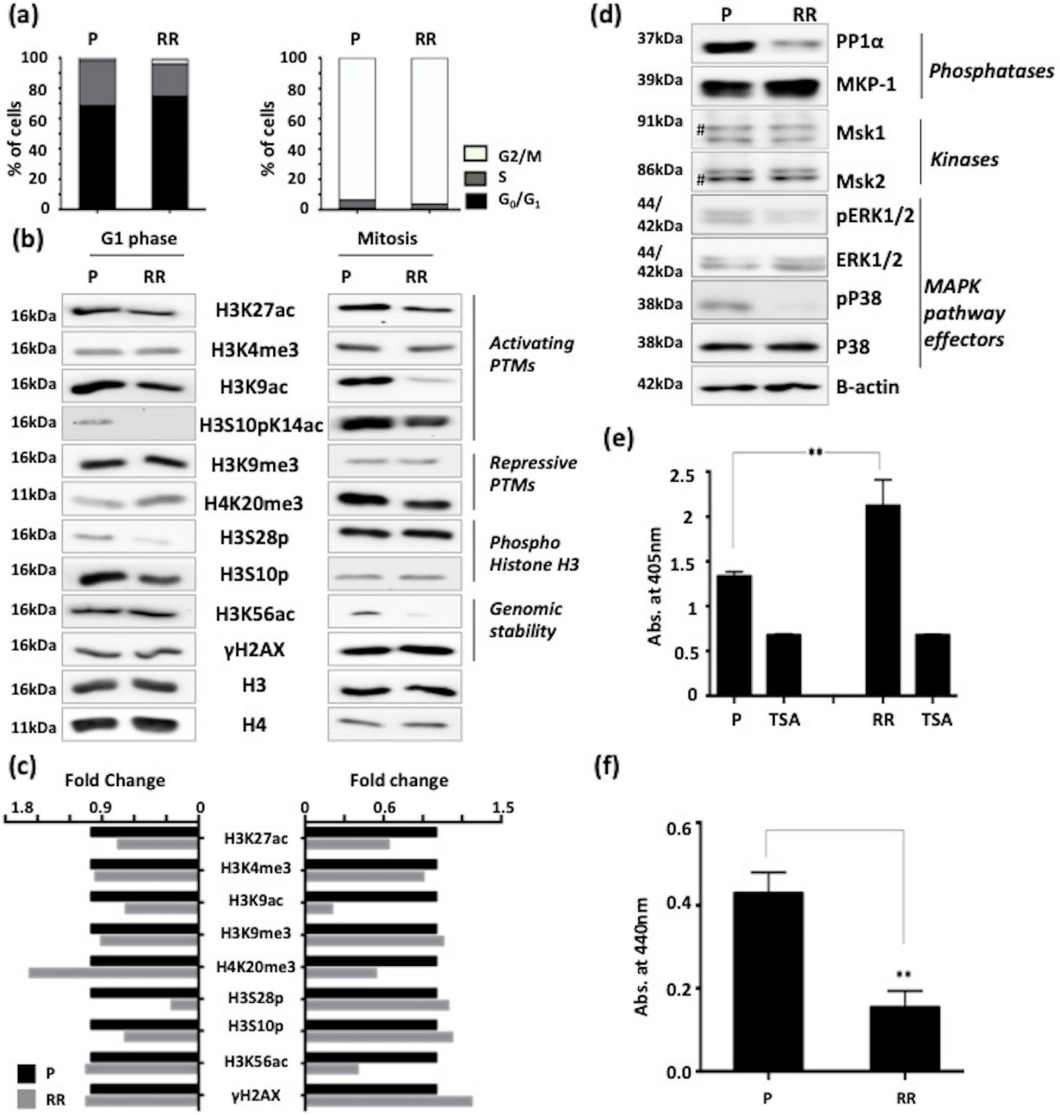
Radioresistant cells have global hypo-acetylation and altered HAT and HDAC activities. **a** Flow cytometry based cell cycle profile of P and RR synchronized in G_0_/G_1_ phase and mitosis. **b** Western blots of site-specific histone PTMs in P and RR synchronized in G_0_/G_1_ and mitotic phases of cell cycle. Western blotting was performed using acid-extracted histones from P and RR (**c**) Densitometry based analysis of western blots. Fold change was calculated after normalizing intensity of RR with P. Fold change of P was taken as 1. **d** Western blots of MAPK pathway proteins in total cell lysates of P and RR. β-actin serves as loading control. **e** Graph depicting comparison of HDAC activity between P and RR. Readout of HDAC activity was measured at 405 nm as a colorimetric reaction. TSA depicts negative control consisting of HDAC inhibitor Trichostatin A (**f**) Graph represents comparison of HAT activity between P and RR. Readout of HAT activity was measured at 440 nm as a colorimetric reaction. Parental MCF7 is denoted as “P” and radioresistant cell line is denoted as “RR”. Statistical analysis is done by Student’s *t* test. ^*^p < 0.05, ^**^p < 0.01, #—nonspecific band, Abs.—absorbance, TSA—trichostatin A. Error bars represent ± S.D. of 3 experiments

Chromatin architecture and cell cycle phase strongly influence the DNA damage response. Since mitosis is the most radio-sensitive phase of the cell cycle and G_0_/G_1_ is a relatively more radio-resistant phase, histone PTM profiling was performed on these populations of parental MCF7 and MCF7-RR (Fig. 4a). Transcriptional activation marks like H3K9ac, H3K27 ac, and H3S10pK14ac showed a decrease in MCF7-RR in both G_0_/G_1_ and mitotic phases and a decrease of H3K56ac only in mitosis (Fig. 4b and c). In the case of 231RR cells, a decrease was observed only in the case of H3K9ac and H3K27 ac (Additional file 3f). Out of histone PTMs that mark transcriptional repression, H4K20me3 was elevated in G_0_/G_1_ phase and decreased in mitosis in MCF7-RR. Interestingly, apart from histone acetylation, phosphorylation of sites H3S10 and H3S28 showed a decrease in G_0_/G_1_ phase in MCF7-RR (Fig. 4b, c) but an increase of H3S10p was observed in 231RR (Additional file 3f). Both these sites are modified by kinases and phosphatases that also regulate the Mitogen-Activated Protein Kinase (MAPK) pathway. Decreased levels of MAPK pathway effector proteins (phospho-ERK1/2 and phospho-P38) were concomitant with increased levels of MAP Kinase Phosphatase- 1 (MKP-1), which is a negative regulator of MAPK pathway. However, there was no change in the levels of kinases MSK-1 and MSK-2 (mitogen- and stress-activated protein kinase-1 and 2) in MCF7-RR (Fig. 4d). Intrigued by the previous observations of chromatin condensation and histone hypo-acetylation, the activity of HATs and HDACs was assessed. Interestingly, MCF7-RR cells exhibited an enhanced HDAC activity and a decrease in HAT activity (Fig. 4e and f). In coherence, an increase in HDAC activity was also observed in 231RR, but the HAT activity remained unchanged (Additional file 3g and h). Lysates containing TSA served as negative controls (Fig. 4e and Additional file 3g). An increased expression of HDAC 2 and 8 (belonging to class I HDAC family) was also observed in MCF7-RR cells (Additional file 2d). Thus, during the acquirement of radio-resistance in breast cancer cells, altered activity of HATs and HDACs leads to an overall condensed chromatin state and altered histone phospho-acetylation.

### Histone deacetylase inhibitor VPA causes retention of γH2AX and radio-sensitization of MCF7-RR cells

**Fig. 5:**
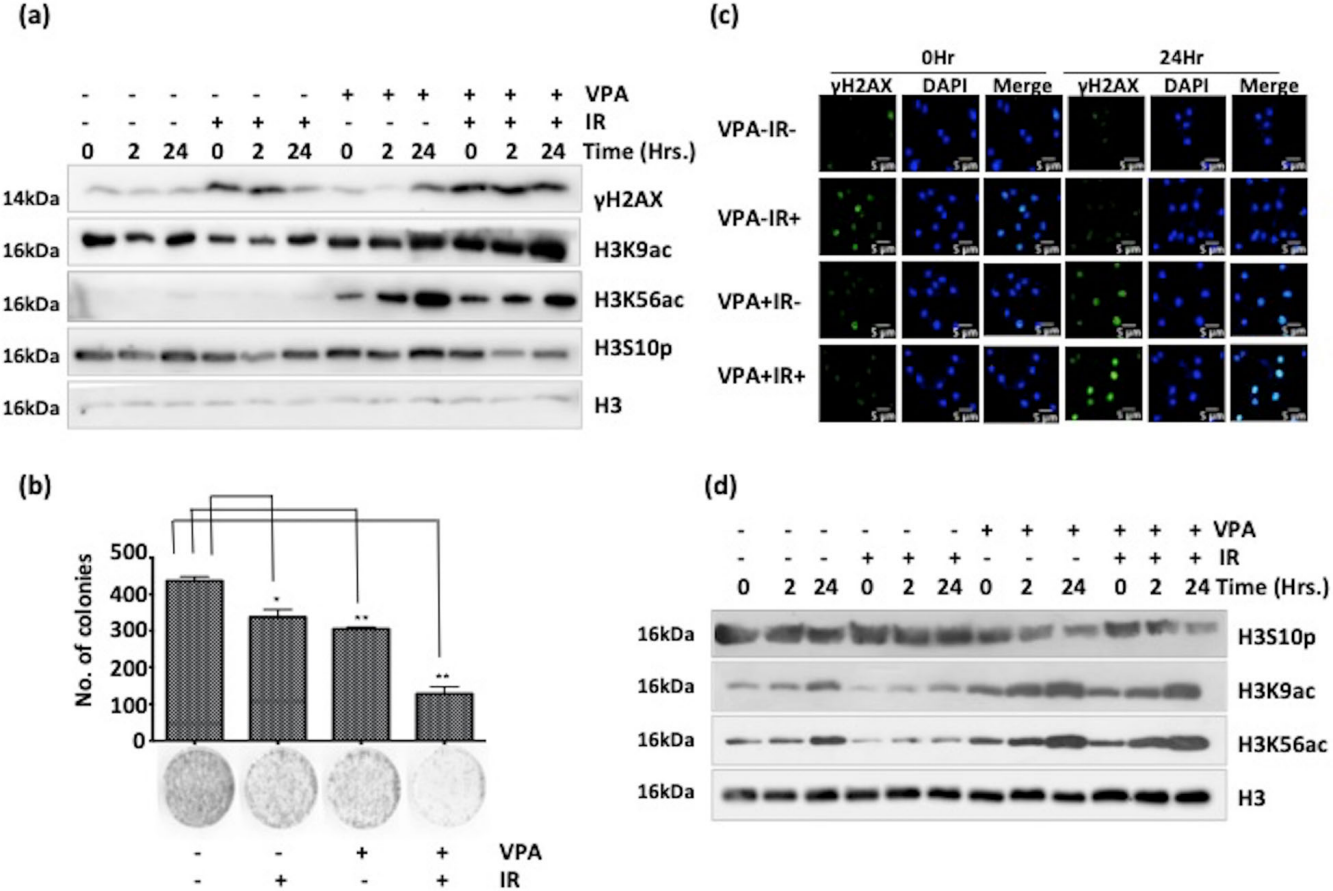
HDAC inhibitor Valproic acid causes retention of γH2AX in both acquired and inherently radioresistant cells. **a** Western blotting depicts changes in levels of site-specific histone PTMs in MCF7-RR at different time points after treatment with 2.5 mM of HDAC inhibitor VPA and 4Gy radiation exposure. **b** Clonogenic assay of MCF7-RR cells to assess radio-sensitization potential of valproic acid. The graph depicts the number of colonies. Cells were treated with 4 Gy radiation and 2.5 mM VPA. **c** Representative z-stack projection images for comparison of retention of γH2AX levels by immunofluorescence in the U87 cell line. Cells were treated with 2.5 mM VPA and exposed to 4 Gy radiation. Scale bar—5 μm. **d** Western blotting for site-specific histone PTMs post irradiation in the U87 cell line. Scale bar—20 μm. Images were processed using LSM browser software. Hrs.—hours, IR—ionizing Radiation, VPA—valproic acid. Statistical analysis is done by Student’s *t* test. ^*^p < 0.05, ^**^p < 0.01. Error bars represent ± S.D. of 3 experiments

The MCF7-RR cells showed an elevated HDAC activity; thus, they were considered to be potential targets for HDAC inhibitor–based radio-sensitization. Both acquired radio-resistant MCF7-RR as well as an intrinsically radio-resistant glioblastoma cell line (U87) were treated with VPA for radio-sensitization. VPA was used at a dose of 2.5 mM, which had no effect on cell proliferation of MCF7-RR (Additional file 4a) and cell cycle progression (with or without radiation) in both the cell lines (Additional file 4b and d). Pre-treatment with VPA was done 2 h before radiation. An elevation of histone acetylation marks like H3K9ac and H3K56ac indicate successful VPA treatment in both cell lines (Fig. 5a, d). Persistence of γH2AX up to 24 h was observed in MCF7-RR and U87 cells subjected to a combinatorial treatment of VPA and radiation (Fig. 5a, c). This indicated delayed DNA repair kinetics compared with cells that received only radiation. Combination treatment of VPA and IR in MCF7-RR caused enhanced cell death, as assessed by clonogenic assay (Fig. 5b). However, the assay also revealed that the 2.5 mM VPA dose, which had not caused cytotoxicity in short-term proliferation (Additional file 4a), was found to be cytotoxic for long-term survival (Fig. 5b). In both MCF7-RR and U87 cell lines, there was only partial recovery of H3S10p post irradiation and VPA treatment (Fig. 5a, d). Additionally, there was also an increase in the levels of MKP-1 phosphatase post VPA treatment in case of MCF7-RR (Additional file 4c). This indicated the possibility of a cross-talk between histone acetylation and phosphorylation in the context of H3S10p recovery post VPA treatment and radiation.

### Variation in the levels of HDAC activity indicates inter-tumoral epigenetic heterogeneity

**Fig. 6:**
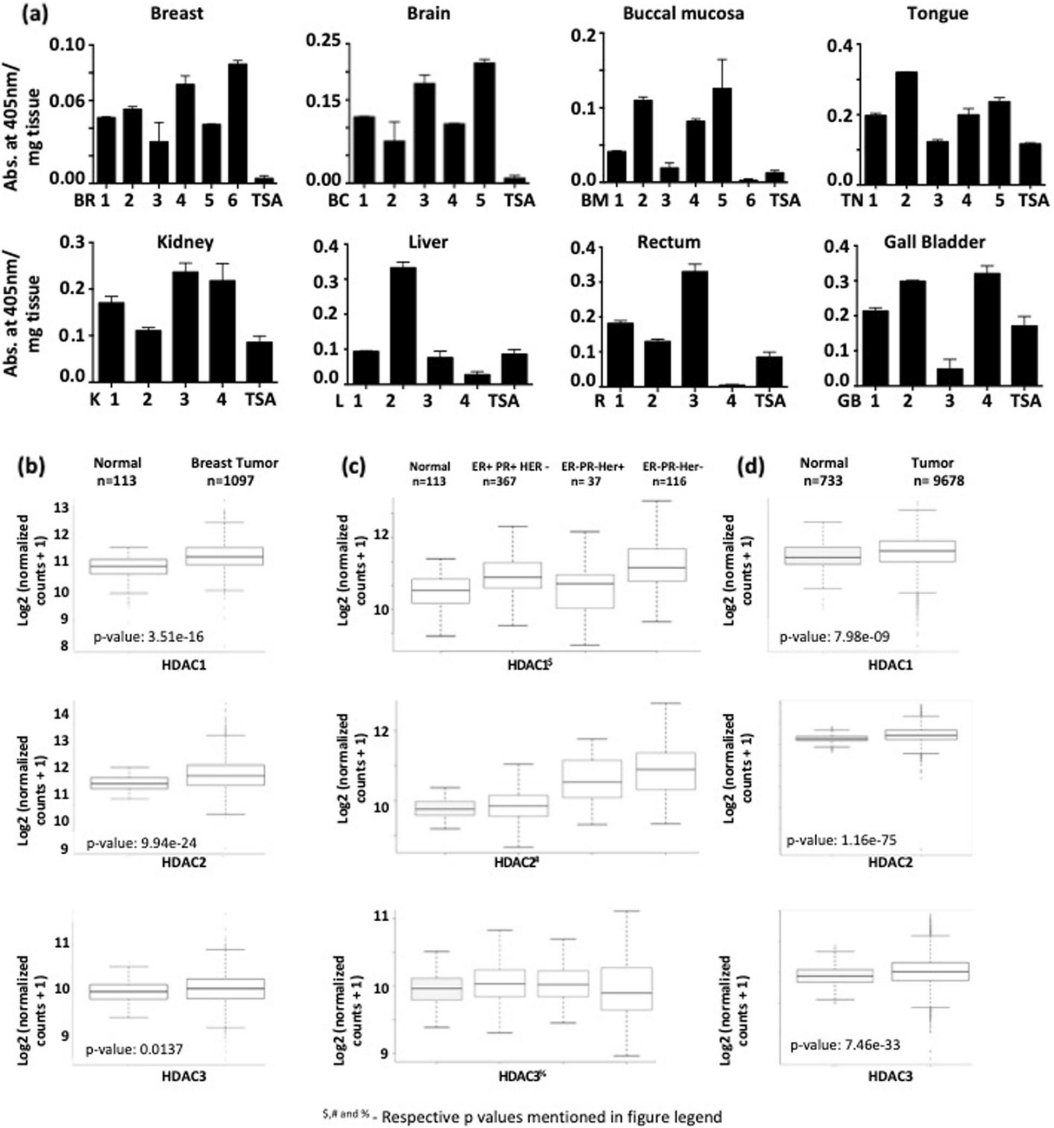
Inter-tumoral variation in levels of HDAC activity and expression emphasizes the need for patient stratification. **a** Graphs depict the HDAC activity in 38 human tumor tissue samples from 8 different tissues. HDAC activity is depicted as absorbance at 405 nm per mg of lysate. TSA indicates negative control consisting of HDAC inhibitor Trichostatin A. Numbers on the X-axis indicate sample number. **b**–**d** Graphs depict expression of HDAC1–3 analyzed from RNA-seq data available in TCGA for (**b**) normal versus breast cancer (**c**) breast cancer subtypes based on hormone classification and (**d**) normal versus pan-cancer. *p* value was determined using the Wilcoxon–Mann–Whitney test analysis. Error bars represent quartile range 25 and 75% respectively for all the samples. Dots represent outliers. BR—breast, BC–brain, BM–buccal mucosa, TN–tongue, K–kidney, L–liver, R–rectum and GB–gall bladder. ^$^*p* values for HDAC1 (by one-way ANOVA) is < 0.0001 and by the Wilcoxon–Mann–Whitney test for individual groups ER + PR + Her- v/s ER-PR-Her+ is 0.0274, ER + PR + Her- v/s ER-PR-Her+  is 0.000191 and ER + PR + Her- v/s ER-PR-Her- is 0.000305. ^#^ *p*-values for HDAC2 (by one- way ANOVA) is < 0.0001 and by Wilcoxon–Mann–Whitney test for individual groups ER + PR + Her- v/s ER-PR-Her+ is 2.71 e-^07^, ER + PR + Her- v/s ER-PR-Her+ is 0.0485 and ER + PR + Her- v/s ER-PR-Her- is 1.85 e^− 23^. ^%^ p-values for HDAC3 (by one- way ANOVA) is 0.4797 and by Wilcoxon–Mann–Whitney test for individual groups ER + PR + Her- v/s ER-PR-Her+ is 0.591, ER + PR + Her- v/s ER-PR-Her+ is 0.738 and ER + PR + Her- v/s ER-PR-Her- is 0.231

**Fig. 7:**
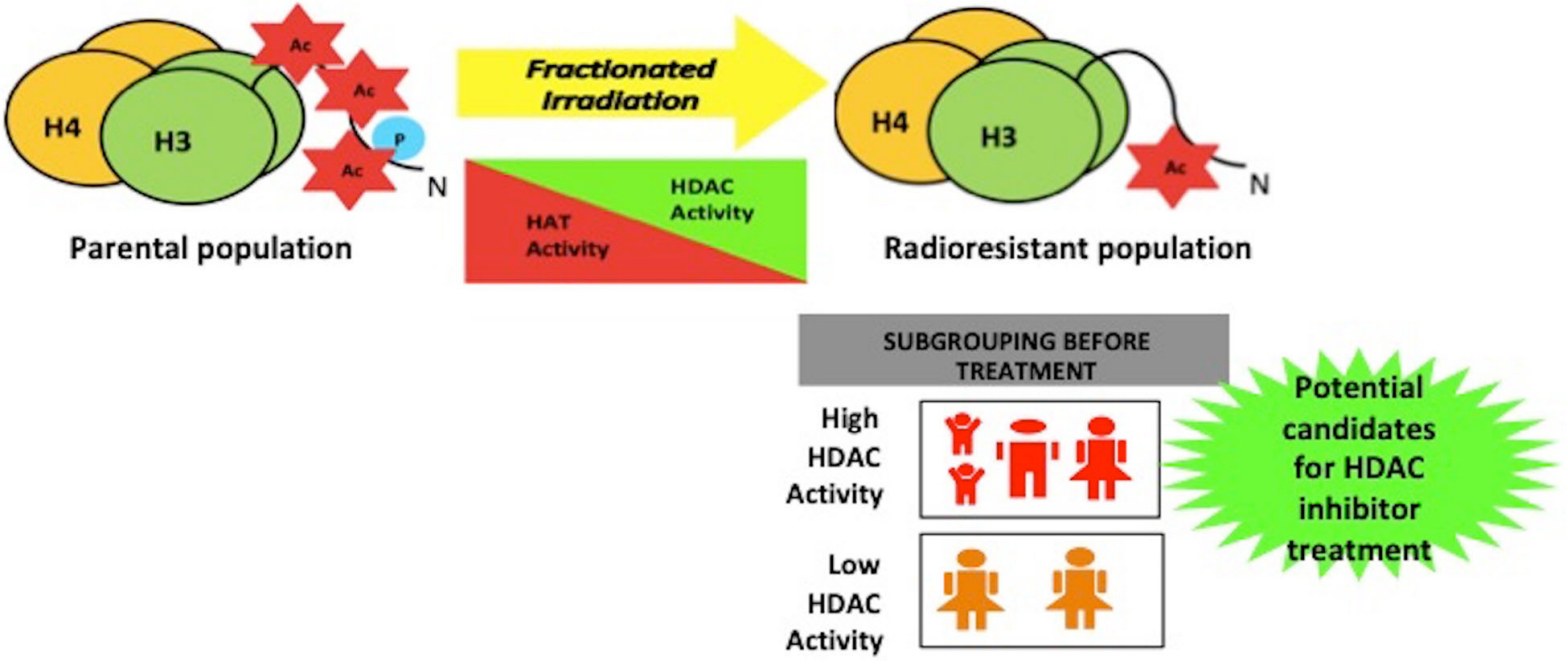
Model depicting epigenetic alterations during radiotherapy, and how an understanding of these changes would be beneficial for patient subgrouping in clinics

Treatment with VPA was able to induce γH2AX retention in both acquired and inherently radio-resistant cell lines. However, reports suggest an ineffectiveness of HDAC inhibitors across various solid tumors [36, 37]. One of the reasons for such a poor response to HDAC inhibitors could be epigenetic heterogeneity within a population. We propose that to maximize the benefit of epi-drug therapy, it is important to choose the correct patients for treatment. Therefore, to investigate inter-tumoral epigenetic heterogeneity in a patient population, HDAC activity was assessed across 8 human tumor types viz. breast, brain, tongue, buccal mucosa, kidney, rectum, gall bladder, and liver. Tumor types were chosen based on the incorporation of radiotherapy in their treatment regime. Histological validation for the tissue of origin and tumor quality/content assessment of the tumor was performed by H&E staining by a pathologist (Additional file 5a-h and Table 1). Interestingly, HDAC activity varied even across patients with similar tumor types, irrespective of tissue of origin (Fig. 6a and Table 1). TSA served as the negative control. Analysis of RNA-seq datasets available in TCGA for breast cancer revealed that indeed, the expression of HDAC 1 and 2 varied between normal and breast cancer samples (Fig. 6b). An additional analysis of HDAC1–3 expression for hormone receptor–based characterization of breast cancer also revealed significant alterations in expression of HDAC 1 and 2, but not HDAC3 (Fig. 6c) across various subtypes. A pan-cancer analysis revealed significant upregulation of HDACs 1–3 across a variety of cancer types (Fig. 6d). Our results suggest that epigenetic modifiers like HDACs show altered activity as well as variable expression in a patient population. Therefore, it might be helpful to obtain information about the epigenetic background of the patient before treatment with epigenome-targeting drugs. Hence, classification of patients into HDAC high and low categories could lead to optimum utilization of HDAC inhibitors as radio-sensitizers and enhance the efficacy of radiation in poor-responders (Fig. 7).

**Table 1:**
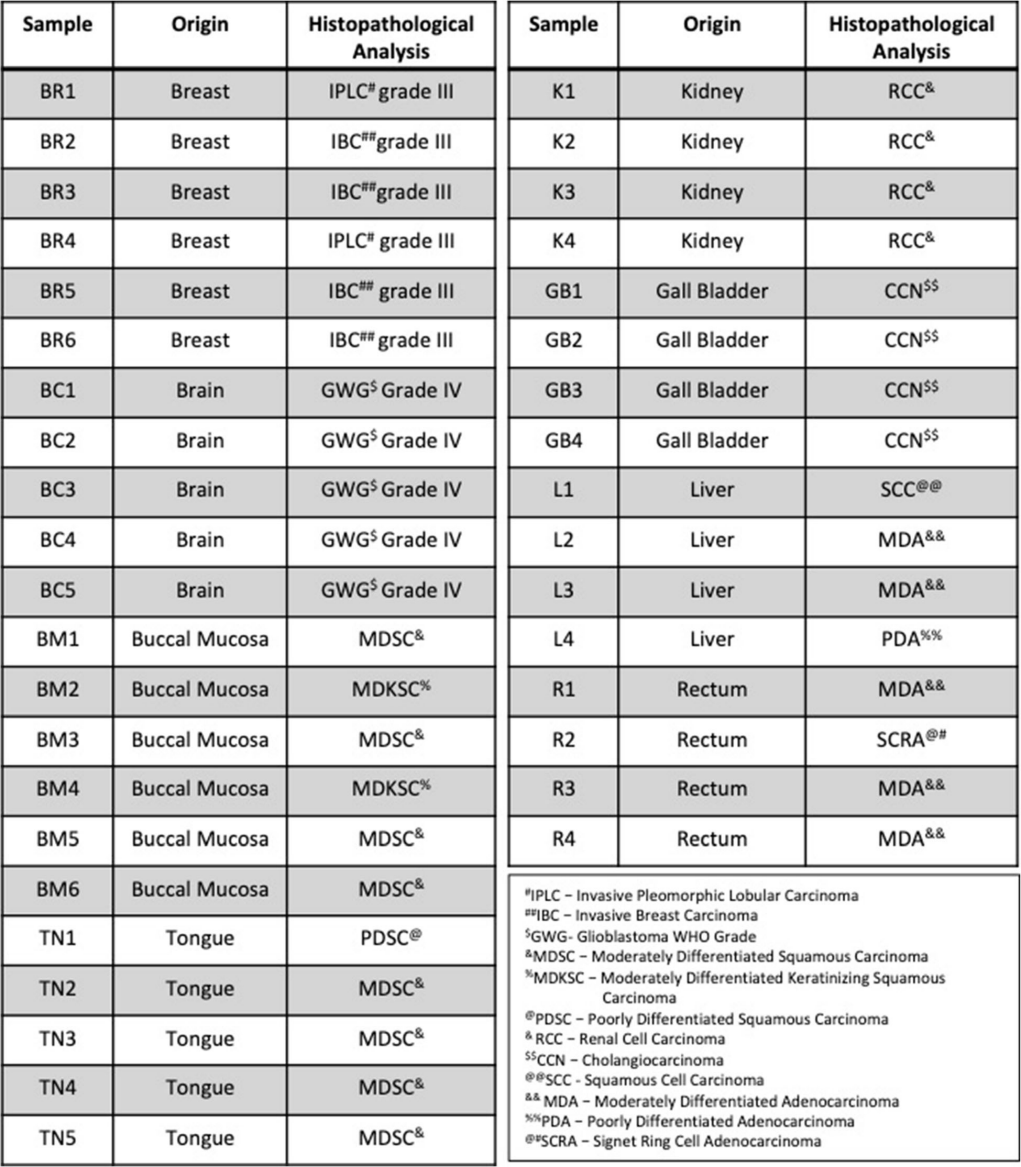
Description of Histopathology of tumor samples used in the study

*BR* -breast, *BC* -brain, *BM* -buccal mucosa, *TN* -tongue, *K* -kidney, *L* -liver, *R* -rectum, *GB* -gall bladder

## Discussion

The emphasis of this study has been to explore the epigenetic alterations that occur as breast cancer cells acquire radio-resistance. Proper application of this understanding can be immensely helpful to develop effective radio-sensitization strategies. To the best of our knowledge, this is the first report demonstrating that altered HAT-HDAC activity, decreased global histone phospho-acetylation, and chromatin condensation are key features of radio-resistant breast cancer cells. The heterogeneity in inter-tumoral HDAC activity of human tumor samples might explain the poor response of HDAC inhibitors in clinics, suggesting their prudent usage after taking into account patient-to-patient epigenetic variation.

Earlier studies on radio-resistant prokaryotes and mammalian cells point towards alteration in the nuclease sensitivity of cells post radiation [38–43]. Indeed, we also observe an increase in heterochromatinization over the course of acquiring radio-resistance in both MCF7 and MDA-MB231 cell lines. Since the euchromatic regions of the genome are more prone to undergo DNA damage post irradiation [21, 44, 45], an increase of overall heterochromatin content may be advantageous for radio-resistant cells. Our report also provides an analysis of the histone PTM alterations that take place during the course of radiotherapy. Histone phosphorylation and acetylation form a “histone code” for immediate-early gene transcription. Our group and others have demonstrated a decrease in the level of site-specific histone phosphorylation and acetylation in response to DNA damage [26, 46]. These reports describe the transient changes of histone PTMs at specific time points post radiation. However, this does not reflect the long-term implications of such alterations during processes like radio-resistance acquirement.

Cell cycle phase is a crucial factor that influences histone PTM levels and also intrinsic cellular radio-sensitivity. It is interesting to note that in radio-resistant MCF7 cells, the decrease of transcription activation histone marks H3K9ac, H3K27 ac and H3S10pK14ac occurs independent of cell cycle phase, indicating that these events are associated with the acquirement of radio-resistance. Histone PTM alterations also influence the gene expression pattern and cellular signaling and thereby, may potentially influence processes like acquired radio-resistance. H3S10p and S28p are both a part of the two “ARKS” motifs present in histone H3 N-terminal tail and are governed by similar kinases and phosphatases, but under contexts of cellular transcription and mitosis [47–49]. Similar to H3S10p, H3S28p has also been reported to be localized at regions of active promoters [49]. In our study, a decrease in the levels of both H3S10p and S28p, concomitant with a decrease in transcription activation marks, strongly suggests an alteration of gene expression profile in MCF7-RR. Likewise, increased level of H3S10p but reduced H3K9ac and K27 ac in 231RR is a probable indicator of global epigenetic alterations that may lead to aberrant gene expression. Ongoing ChIP-seq based studies may help to elucidate how the decreased histone phospho-acetylation may be important for regulating gene expression during radio-resistance. Additionally, analysis of levels of H3S10p kinases and phosphatases revealed increased levels of MKP-1 phosphatase in MCF7-RR. In the context of cellular signaling, MKP-1 is a negative regulator of the MAPK pathway [50, 51]. We observe an increase in levels of MKP-1, thus explaining the decreased phosphorylation of ERK1/2 and P38. Upregulation of MKP-1 is also implicated to be a poor prognostic factor for breast cancer and mediates therapy resistance to Her2-positive breast tumors [52, 53]. Thus, our report suggests that alteration in levels of histone PTMs and their respective modifying enzymes during radiotherapy might influence gene expression and functioning of key cellular pathways.

Dysregulation of HDACs is implicated in various cancers and plays an important role during DDR [54, 55]. We observed an altered HDAC/HAT activity pattern in radio-resistant breast cancer cells, with an increase in HDAC activity for both MCF7-RR and 231RR. This explains the previously described global histone hypo-acetylation as well as chromatin condensation. Treatment of this target population by HDAC inhibitors could lead to potential sensitization towards radiation. Advantages of using HDAC inhibitor VPA include FDA approval, low cellular toxicity, increased half-life, and the ability to cross the blood–brain barrier. To compare the mechanism of action of VPA, both an acquired (MCF7-RR) and inherently radio-resistant (U87) cell lines were used. Interestingly, we observed a reduction in the levels of H3S10p post VPA and radiation combination treatment in both types of cells. Our group has previously reported that H3S10p undergoes dephosphorylation and subsequent re-phosphorylation in response to DNA damage, both of which are crucial for effective DNA repair and cell survival post radiation [56, 57]. Since HDACs can act on both histone and non-histone substrates, their mechanism of action is varied [27]. Treatment with HDAC inhibitor TSA decreases phospho-histone H3 due to non-localization of kinetochore protein BubR1, potentially attributed to impaired deacetylation of non-histone substrates of HDACs [58]. Also, since histone PTMs function as a “histone code” [59], persistence of histone hyper-acetylation post VPA treatment could affect the phosphorylation of H3S10p. A study suggests that hypo-acetylated histone tails are preferred substrates for Aurora Kinase B [60]. We observe an induction in the levels of MKP-1 phosphatase post VPA treatment, concomitant with a previous study [61]. But there have been no reports about histone kinases as substrates of HDACs. Hence, the reduced levels of H3S10p after VPA treatment may be attributed to both histone hyper-acetylation and the effect of VPA on non-histone substrates, which may affect survival of cells post-radiation.

Despite showing promising results in hematological malignancies, the utility of HDAC inhibitors in solid tumors has been limited due to toxicity, poor pharmacokinetics as well as low half-life. Our group has recently demonstrated HDAC activity in serum of human normal and tumor counterparts [62]. This suggests that monitoring HDAC/HAT activities during treatment may provide “real-time” information about treatment response. In this study, we observe an increase in HDAC activity during acquired radio-resistance. Therefore, we propose that an evaluation of tumor HDAC activity be carried out before beginning with radiotherapy, which may help to identify suitable candidates for HDAC inhibitor–based radio-sensitization. This is based on our observation of epigenetic heterogeneity in terms of HDAC activity status across 38 tumor samples. It is very interesting that even in our small sample size, tumors of the same histopathological type display variation in HDAC activity, for example, BR3 and BR6 are both invasive breast carcinoma grade III cancers, yet have more than two-fold difference in HDAC activity. This points towards the extensive inter-tumor heterogeneity in a population, emphasizing that not all patients may respond similarly to HDAC inhibitor therapy. Additionally, we were able to check the HDAC activity of as little as 1 mg of tumor tissue, thereby excluding the limitation of sample amount and availability from a biopsy. Therefore, subgrouping of patients into low and high HDAC activity groups, and then treatment of the correct subgroup may help to greatly increase the efficacy of HDAC inhibitor as a radio-sensitizer. According to our analysis, patients having tumors with high HDAC activity are the suitable group for HDAC inhibitor–based radio-sensitization. However, altered HDAC activity may not be the only measure for poor response towards radiation. In such a scenario, it is possible that other factors like epigenetic/genetic determinants, tumor microenvironment and stemness might impart radio-sensitivity [14–17]. Therefore, studies are required to understand the cause of poor response to radiation in cancer patients. Such an understanding of epigenetic alterations during radiotherapy may help in preventing episodes of recurrence due to radio-resistance. Therefore, the need of the hour is to realize the tremendous potential of epi-drug therapy and utilize strategies like patient subgrouping for better disease management.

## Conclusion

To summarize, our study demonstrates that epigenetic alterations are an important player during radiotherapy and may be responsible for acquired radio-resistance. Radio-resistant cells show high HDAC activity–low HAT activity that explains the compact chromatin architecture and altered histone phospho-acetylation. Also, epigenetic variation across human tumor samples in terms of the tumor HDAC activity indicates that prior stratification of patients is important before beginning any HDAC inhibitor–based treatment.

## Methods

### Cell culture, synchronization, and irradiation

The cell lines used in this study are epithelial origin breast cancer (MCF7 and MDA-MB231) and glioblastoma (U87) cell lines. All cell lines were cultured in DMEM media (Invitrogen) supplemented with 2 mM glutamine (Sigma), 10% fetal bovine serum (FBS) (Gibco) and antibiotic antimycotic solution (Himedia). Cells were maintained at 37 °C and 5% CO_2_. Synchronization in G_0_/G_1_ and mitotic phase of the cell cycle was done by serum starvation using 0.02% serum for 72 h and 18 h incubation with 200 ng/ml nocodazole (Sigma), respectively. Cells were irradiated using Co-60 radioactive source–based machine Bhabhatron-II (Panacea Medical Technologies Ltd. and Bhabha Atomic Research Centre (BARC), India) installed at the Department of Radiation Oncology, ACTREC. Field size was 25 cm × 25 cm, source-to-skin distance (SSD) as 80 cm, and gantry was angled at 180° to the specimen.

### Generation of radio-resistant cell line

Fractionated Irradiation (FIR) was used to generate radio-resistant cells. Ten fractions of 2 Gy each were used to generate MCF7-RR and 231RR, respectively. Cells were maintained up to 60% confluency at the time of irradiation and exposed to 2-Gy radiation dose/fractions for 10 cycles (Dose rate 0.75 Gy/min). Cells were allowed to recover and gain 80–85% confluency before sub-culturing for the next dose. Parental cells were sham irradiated during the process. Cells were allowed to grow for 21 days after the final dose of 20 Gy. The radio-resistant populations are designated as parental MCF7-RR and 231RR.

### Clonogenic assay

Cells were irradiated, allowed to recover for 6 h, counted, seeded and maintained for 14 days. Colonies were fixed with 4% paraformaldehyde (Sigma) for 20 min followed by washing with phosphate buffered saline (PBS). Staining was performed using 0.5% crystal violet. Colonies were counted under a light microscope and only colonies containing > 50 cells were considered to be viable. The plating efficiency was calculated as mentioned [63] and surviving fraction of each cell line was calculated. The D_0_ value of each cell line was calculated using the survival fraction and the doses 2 Gy and 4 Gy.

### Cell migration assay

Cells were seeded in a 6-well plate, allowed to grow for 24 h and then serum starved in 0.02% FBS containing media for 72 h. At the time of the experiment, 3 wounds were made in the 100% confluent cell monolayer. Debris of dislodged cells was washed off using PBS and 0.02% serum-containing media was added. Cells were incubated in a CO_2_ chamber at 37 °C and monitored for migration using live-cell microscopy for 20 h.

### Cell proliferation assay

A total of 1000 cells of parental MCF7 and MCF7-RR were seeded in a 96-well plate and growth was analyzed for 96 h. A 3-(4,5-dimethylthiazol-2-yl)-2,5-diphenyltetrazolium bromide (MTT) reagent (5 mg/ml in PBS, Sigma) was added to each well at 1/10th medium volume and incubated for 4 h. Formazan crystals were solubilized using MTT solubilization buffer (10% sodium dodecyl sulfate (SDS), 0.01 M HCl) and incubated for 24 h in dark. Absorption at 570 nm was measured using Spectrostar Nano Biotek LabTech 96-well plate reader.

### Flow cytometry–based cell cycle analysis

Cell cycle analysis was carried out using propidium iodide (PI)-based DNA content analysis as described previously [26]. DNA content analysis was carried out using fluorescence-activated cell sorting (FACS) Calibur flow cytometer (Becton Dickinson) and analysis done using MODFIT software by Verity house.

### AnnexinV/PI staining

The apoptotic population analysis was done by AnnexinV- Fluorescein isothiocyanate (FITC) apoptosis detection kit (Sigma), strictly following manufacturer instructions. Fluorescence was immediately measured using FACS Calibur flow cytometer (Becton Dickinson) and analysis done using CELLQUEST software.

### Transmission Electron microscopy

Parental MCF7 and MCF7-RR cell pellets were fixed with 3% glutaraldehyde. Post-fixation was performed with 1% osmium tetraoxide. Alcoholic uranyl acetate treatment for 1 min and lead citrate treatment for 30 s was done for grid contrasting and then observed under Carl Zeiss LIBRA120 EFTEM.

### Raman spectroscopy

Fresh cell pellets of parental MCF7 and MCF7-RR were fixed using 1% Paraformaldehyde (PFA) at 4 °C for 10 min followed by two washes of saline at 5000 rpm for 5 min at 4 °C. A total of 30 spectra were analyzed for both parental and radio-resistant MCF7. Raman spectra were recorded using a commercial Raman micro spectroscope (WITec alpha300RS, λ_X_-532 nm, 10 mW, 600 grooves/mm). Preprocessed Raman spectra (smoothening, fifth point, baseline, fifth order, and vector normalization) in 650–1750 cm^− 1^ were subjected to PCA and PCA-LDA using commercial Unscrambler® X software.

### Micrococcal nuclease digestion assay

MNase digestion was performed as described previously [26]. The DNA pellet was dissolved in 50 μl Tris-EDTA (TE) buffer and samples were resolved on 1.8% 1× tris-EDTA-acetic acid (TAE) agarose gel containing 0.5 μg/ml ethidium bromide. The image was analyzed using Image J software.

### Histone isolation

Histone isolation was performed as described earlier [26]. The chromatin bound histones were extracted using acid extraction method. The histones obtained in the final pellet was resuspended in 0.1% β-mercaptoethanol and stored in − 20 °C.

### Western blotting

Histones and total cell lysates were resolved on 18% and 10% SDS-poly-acrylamide gel electrophoresis (SDS-PAGE) respectively, transferred on PVDF membrane and subjected to western blotting. Antibodies and their dilution used are as provided in Additional file 6.

### Quantitative PCR

RNA extraction from parental and MCF7-RR was done by Trizol method, followed by DNaseI treatment (Fermentas) and cDNA synthesis using random hexamer primers (Revert-Aid cDNA Synthesis Kit, Thermo Scientific), strictly as per manufacturer’s instructions. Real-time PCR was performed using gene-specific primers (Additional file 7) using amplification conditions of 30 s at 94 °C, 1 min at 60 °C, and 1 min at 72 °C for 30 cycles followed by 10 min of final extension. SYBR-Green from Applied Biosystems was used for real-time PCR. The reactions were performed and monitored using Quant-Studio 12 K Flex Real-Time PCR System. Fold change was calculated using the ΔΔCt method. The expression levels of MCF7-RR were plotted as fold change normalized to MCF7 parental cell line.

### Immunofluorescence microscopy

Immunofluorescence was performed as described previously [26]. The specific dilutions used for antibodies are described in Additional document 4. PKH staining (Sigma)–based live-cell staining for cell morphology visualization was performed according to manufacturer’s instruction. Imaging was done using Zeiss 510 meta confocal microscope.

### Human tissue sample collection

Approval from Institute Ethics Committee III (Project number 164) was obtained for working on human tumor samples, collected retrospectively with an approved waiver of consent. Samples were collected from the Biorepository of ACTREC-TMC. Histopathological analysis of the samples confirmed tumor status and quality. Samples were stored in − 80 °C and used as required.

### HDAC and HAT activity assay

Assays were performed using the colorimetric HDAC and HAT activity assay kits from BioVision (BioVision Research Products, USA) as per manufacturer instructions. A total of 100 μg of cell lysate was used for the assays. The HDAC activity of human tumor samples was assessed from tissue samples powdered in liquid nitrogen. Fifty micrograms of tissue powder was dissolved in 300 μl RIPA buffer (250 mM sucrose, 50 mM Tris-Cl, pH 7.5, 25 mM KCl, 5 mM MgCl2, 0.2 mM PMSF, 50 mM NaHSO3, 45 mM sodium butyrate, 10 mM β-ME, 0.2% TritonX-100) and incubated on ice for 30 min, followed by centrifugation at 5000 rpm for 10 min. The pellet obtained was sonicated (5 s using 30% amplitude) and centrifuged at 15000 rpm for 30 min. Supernatant equivalent to 1 mg tissue (6 μl) was used for HDAC assay. Each tissue sample had its respective blank, i.e., without lysine developer. Absorbance was estimated using a 96-well plate reader at 405 nm and 440 nm for HDAC and HAT assays, respectively.

### In silico analysis in TCGA pan-cancer dataset

To analyze the expression levels of HDAC1, HDAC2, and HDAC3 in normal and tumor samples of pan-cancer, TCGA PANCAN normalized RSEM counts were obtained from the UCSC cancer genome browser. The TCGA PANCAN study includes multiple cancers, listed as follows: acute myeloid leukemia, adreno-cortical carcinoma, bladder urothelial carcinoma, lower grade glioma, breast invasive carcinoma, cervical squamous cell carcinoma and endo-cervical adenocarcinoma, cholangiocarcinoma, colorectal adenocarcinoma, esophageal carcinoma, glioblastoma multiforme, head and neck squamous cell carcinoma, kidney chromophobe, kidney renal clear cell carcinoma, kidney renal papillary cell carcinoma, liver hepatocellular carcinoma, lung adenocarcinoma, lung squamous cell carcinoma, lymphoid neoplasm diffuse large B-cell lymphoma, mesothelioma, ovarian serous cystadeno-carcinoma, pancreatic adenocarcinoma, pheochromocytoma and paraganglioma, prostate adenocarcinoma, sarcoma, skin cutaneous melanoma, stomach adenocarcinoma, testicular germ cell tumors, thyroid carcinoma, uterine carcino-sarcoma, uterine corpus endometrial carcinoma, and uveal melanoma. These counts were transformed in (Log_2_ + 1) values and represented between normal (*n* = 677) and tumor samples (*n* = 9078). R3.3.3 software (http://www.R-project.org/) was used for boxplot representation. *p* value was determined using Wilcoxon–Mann–Whitney test analysis and one-way ANOVA (for hormone-based subgrouping of breast cancer).

### Statistical analysis

All numerical data were expressed as average of values obtained ± standard deviation (SD). Statistical significance was determined by conducting a Student’s *t* test.

## Supplementary information

**Additional file 1. **(a) Clonogenic assay depicting enhanced cell survival of parental MCF7, 10Gy and 20Gy radioresistant cells at different radiation doses. (b) Graph depicting D_0_ values of MCF7 parental, 10Gy and 20Gy radioresistant populations. (c) Representative images of flow cytometry based analysis of AnnexinV and Propidium Iodide positive population. (d) Representative images of changes in cell migration potential of radioresistant MCF7 and MCF7-RR, assessed by live cell microscopy. Parental MCF7 is denoted as “P” and radioresistant cell line is denoted as “RR”. Statistical analysis is done by student’s t-test. *n* = 3 for all experiments. ^*^p < 0.05, ^**^p < 0.01. n.s.- not significant. Error bars represent ± S.D. of 3 experiments.

**Additional file 2. **(a) Representative z-stack projection images for immunofluorescence analysis of P and RR depicting changes in organization of α-tubulin. Magnification – 40x, scale bar- 10 μm. (b) Representative z-stack projection images for immunofluorescence analysis of P and RR depicting change in cellular morphology by PKH staining. Magnification – 40x, scale bar- 10 μm. (c) Graph depicting comparison of nuclear area between P and RR. Area was quantified from *n* = 50 DAPI stained nuclei. (d) Real time PCR based analysis depicts alteration in expression of different HDAC genes. Expression normalized to MCF7-parental. Fold change 1 depicts levels of parental MCF7. Images were processed using LSM browser software. Parental MCF7 is denoted as “P” and radioresistant cell line is denoted as “RR”. Statistical analysis is done by student’s t-test. n = 3 for all experiments. ^*^p < 0.05, ^**^p < 0.01 and a.u.- arbitrary units. Error bars represent ± S.D. of 3 experiments.

**Additional file 3.** (a) Clonogenic assay depicting enhanced cell survival of 231P and 231RR at different radiation doses. (b) Graph depicting number of colonies obtained after subjecting parental MDA-MB231 and 231RR to 4Gy and 8Gy radiation. (c) Chromatin architecture alterations analyzed by Micrococcal Nuclease (MNase) assay visualized on 1.8% TAE-agarose gel. Time points indicate the duration of incubation of nuclei with MNase. (d) Densitometry based representation of MNase digestion. Red arrows point to areas of overall change in chromatin architecture between 231P and 231RR. (e) Flow cytometry based cell cycle profile of 231P and 231RR, representative of cell cycle profile for all subsequent experiments. (f) Western blots depict levels of histone PTMs in 231P and 231RR. Western blotting was performed using acid extracted histones from P and RR (g) Graph depicting comparison of HDAC activity between 231P and 231RR. Readout of HDAC activity was measured at 405 nm as a colorimetric reaction. TSA depicts negative control consisting of HDAC inhibitor Trichostatin A (h) Graph represents comparison of HAT activity between 231P and 231RR. Readout of HAT activity was measured at 440 nm as a colorimetric reaction. 231P and 231RR represents parental and radio-resistant MDA-MB231 cells, respectively. Statistical analysis is done by student’s t-test. ^*^p < 0.05, ^**^p < 0.01, Abs. – absorbance, TSA – Trichostatin A. Error bars represent ± S.D. of 3 experiments.

**Additional file 4.** (a) Graph represents change in percentage growth of P and RR after 48 hours of dose dependent VPA treatment. (b) Graphical representation of changes in cell cycle profile of RR at different time points upon IR (4Gy) and VPA (2.5 mM) treatment. (c) Western blots depict levels of H3S10p modifying kinases and phosphatases at different time points post VPA treatment and IR exposure in radioresistant cell line. (d) Graph depicts cell cycle profile of U87 cell line at different time points post radiation and VPA treatment. UT- Untreated and Hrs. = Hours. Parental MCF7 is denoted as “P” and radioresistant cell line is denoted as “RR”.

**Additional file 5.** (a-h) Representative images of Hematoxylin and Eosin staining of human tumor samples, assessed by pathologist for tumor content. Samples were derived from tumors of eight different tissue origins. Magnification – 40X, Scale bar- 20 μm.

**Additional file 6.** Details of the antibodies used in the study.

**Additional file 7.** Sequence of primers used in the study.

## Data Availability

The protocols are detailed in the manuscript for scientists wishing to use them for their research work. Also, any supporting data will be made available to editors and peer-reviewers, if required for the purpose of evaluating the manuscript.

## Abbreviations

DDR: DNA damage response
EMT: Epithelial to mesenchymal transition
FIR: Fractionated irradiation
Gy: Gray
HATs: Histone acetyl transferases
HDACs: Histone deacetylases
LET: Linear energy transfer
MNase: Micrococcal nuclease
PIK3CA: Phosphatidylinositol-4, 5-bisphosphate 3-kinase catalytic subunit alpha
PTMs: Post-translational modifications
SSD: Source to skin distance
VPA: Valproic acid
