# Supplementary figures and images for: Elevated HDAC activity and altered histone phospho-acetylation confer acquired radio-resistant phenotype to breast cancer cells
Source: Clin Epigenetics. 2020 Jan 3;12:4. doi: 10.1186/s13148-019-0800-4 (PMC6942324; doi:10.1186/s13148-019-0800-4)

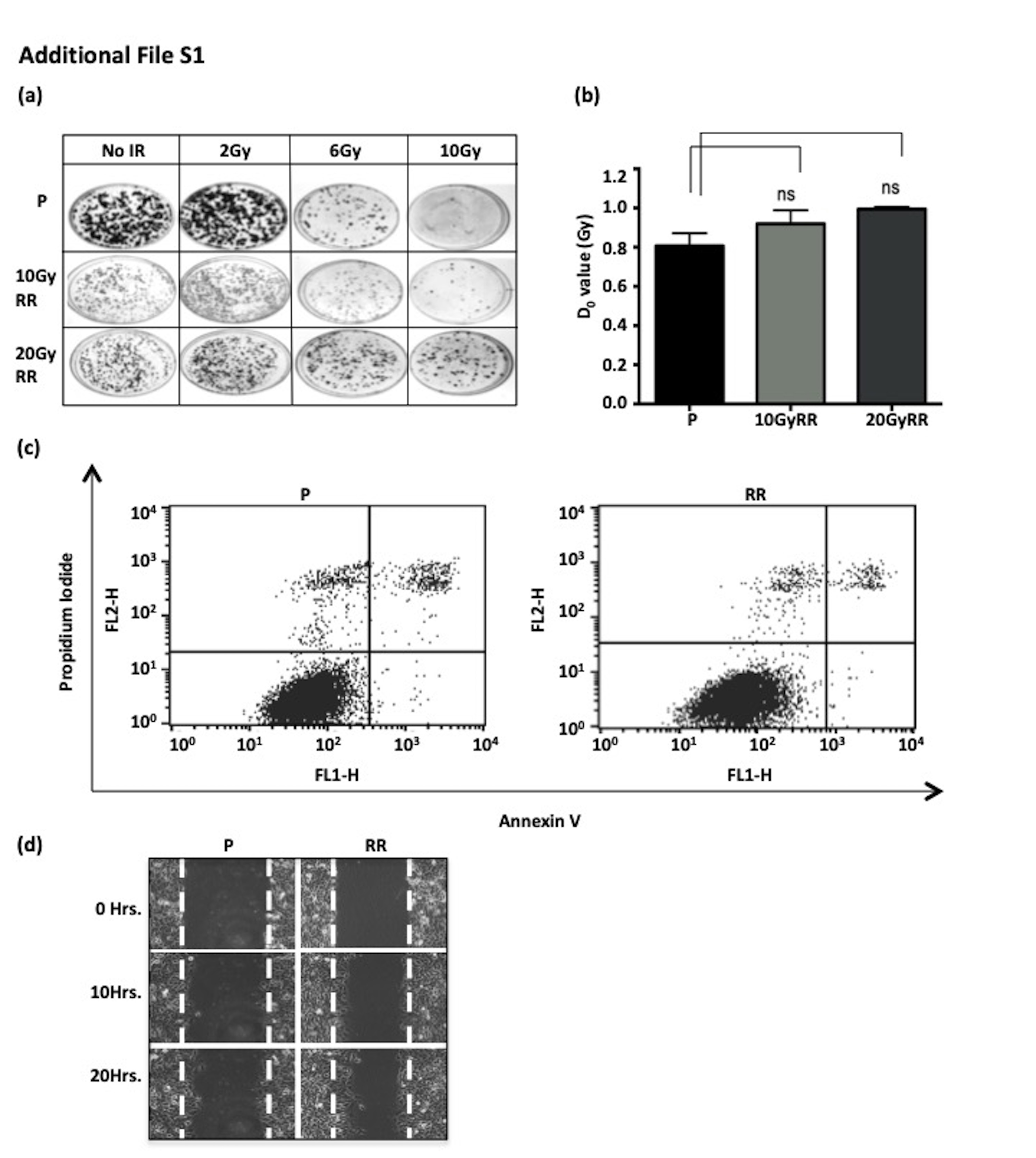

Supplement: Supplementary file 1 — Additional file 1. (a) Clonogenic assay depicting enhanced cell survival of parental MCF7, 10Gy and 20Gy radioresistant cells at different radiation doses. (b) Graph depicting D0 values of MCF7 parental, 10Gy and 20Gy radioresistant populations. (c) Representative images of flow cytometry based analysis of AnnexinV and Propidium Iodide positive population. (d) Representative images of changes in cell migration potential of radioresistant MCF7 and MCF7-RR, assessed by live cell microscopy. Parental MCF7 is denoted as “P” and radioresistant cell line is denoted as “RR”. Statistical analysis is done by student’s t-test. n = 3 for all experiments. *p < 0.05, **p < 0.01. n.s.- not significant. Error bars represent ± S.D. of 3 experiments. [file 13148_2019_800_MOESM1_ESM.jpg]

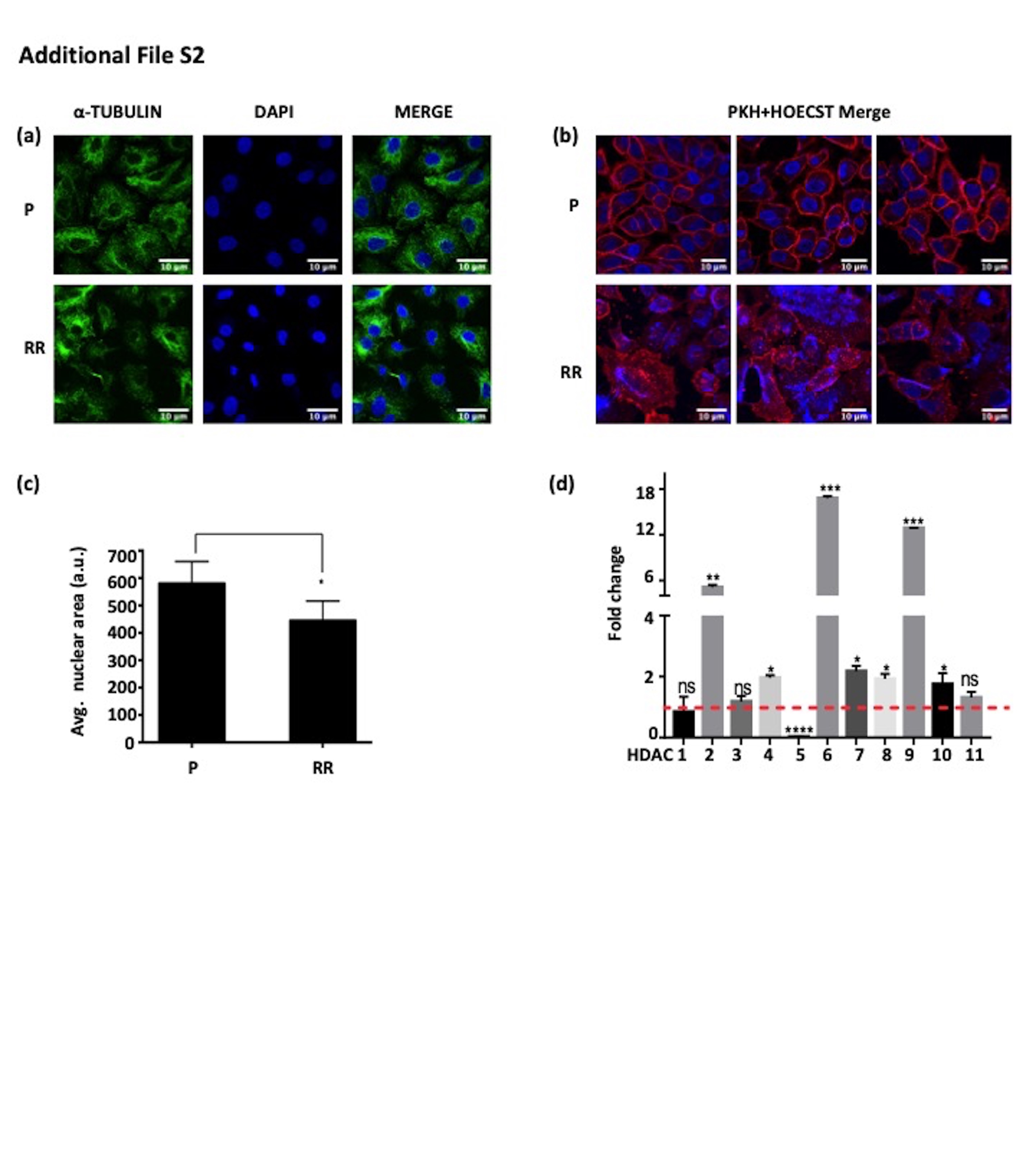

Supplement: Supplementary file 2 — Additional file 2. (a) Representative z-stack projection images for immunofluorescence analysis of P and RR depicting changes in organization of α-tubulin. Magnification – 40x, scale bar- 10 μm. (b) Representative z-stack projection images for immunofluorescence analysis of P and RR depicting change in cellular morphology by PKH staining. Magnification – 40x, scale bar- 10 μm. (c) Graph depicting comparison of nuclear area between P and RR. Area was quantified from n = 50 DAPI stained nuclei. (d) Real time PCR based analysis depicts alteration in expression of different HDAC genes. Expression normalized to MCF7-parental. Fold change 1 depicts levels of parental MCF7. Images were processed using LSM browser software. Parental MCF7 is denoted as “P” and radioresistant cell line is denoted as “RR”. Statistical analysis is done by student’s t-test. n = 3 for all experiments. *p < 0.05, **p < 0.01 and a.u.- arbitrary units. Error bars represent ± S.D. of 3 experiments. [file 13148_2019_800_MOESM2_ESM.jpg]

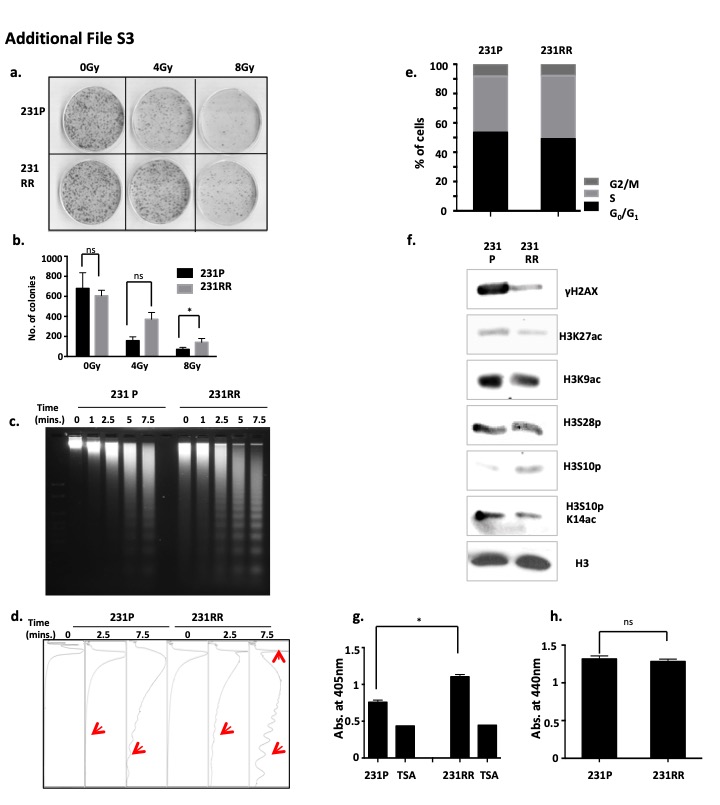

Supplement: Supplementary file 3 — Additional file 3. (a) Clonogenic assay depicting enhanced cell survival of 231P and 231RR at different radiation doses. (b) Graph depicting number of colonies obtained after subjecting parental MDA-MB231 and 231RR to 4Gy and 8Gy radiation. (c) Chromatin architecture alterations analyzed by Micrococcal Nuclease (MNase) assay visualized on 1.8% TAE-agarose gel. Time points indicate the duration of incubation of nuclei with MNase. (d) Densitometry based representation of MNase digestion. Red arrows point to areas of overall change in chromatin architecture between 231P and 231RR. (e) Flow cytometry based cell cycle profile of 231P and 231RR, representative of cell cycle profile for all subsequent experiments. (f) Western blots depict levels of histone PTMs in 231P and 231RR. Western blotting was performed using acid extracted histones from P and RR (g) Graph depicting comparison of HDAC activity between 231P and 231RR. Readout of HDAC activity was measured at 405 nm as a colorimetric reaction. TSA depicts negative control consisting of HDAC inhibitor Trichostatin A (h) Graph represents comparison of HAT activity between 231P and 231RR. Readout of HAT activity was measured at 440 nm as a colorimetric reaction. 231P and 231RR represents parental and radio-resistant MDA-MB231 cells, respectively. Statistical analysis is done by student’s t-test. *p < 0.05, **p < 0.01, Abs. – absorbance, TSA – Trichostatin A. Error bars represent ± S.D. of 3 experiments. [file 13148_2019_800_MOESM3_ESM.jpg]

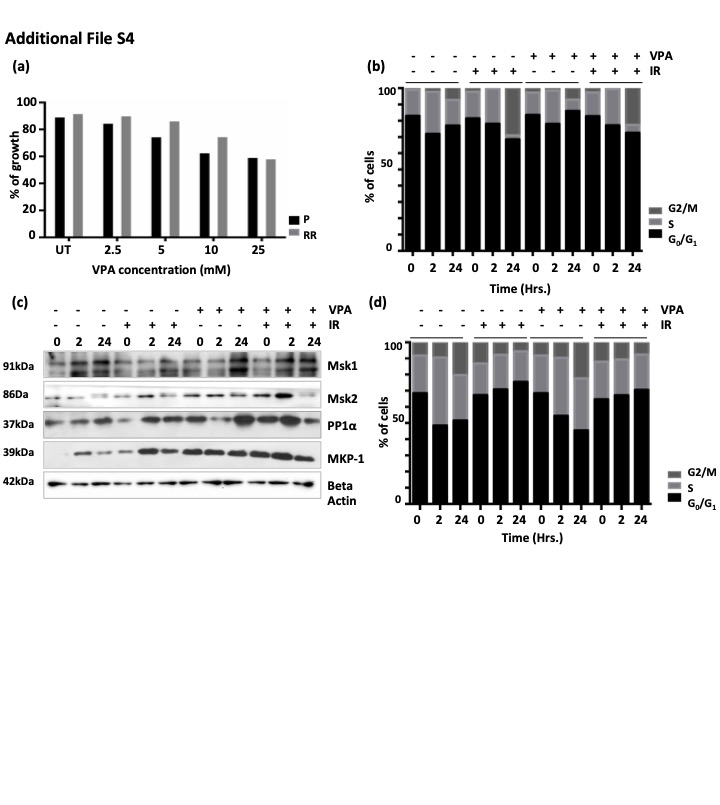

Supplement: Supplementary file 4 — Additional file 4. (a) Graph represents change in percentage growth of P and RR after 48 hours of dose dependent VPA treatment. (b) Graphical representation of changes in cell cycle profile of RR at different time points upon IR (4Gy) and VPA (2.5 mM) treatment. (c) Western blots depict levels of H3S10p modifying kinases and phosphatases at different time points post VPA treatment and IR exposure in radioresistant cell line. (d) Graph depicts cell cycle profile of U87 cell line at different time points post radiation and VPA treatment. UT- Untreated and Hrs. = Hours. Parental MCF7 is denoted as “P” and radioresistant cell line is denoted as “RR”. [file 13148_2019_800_MOESM4_ESM.jpg]

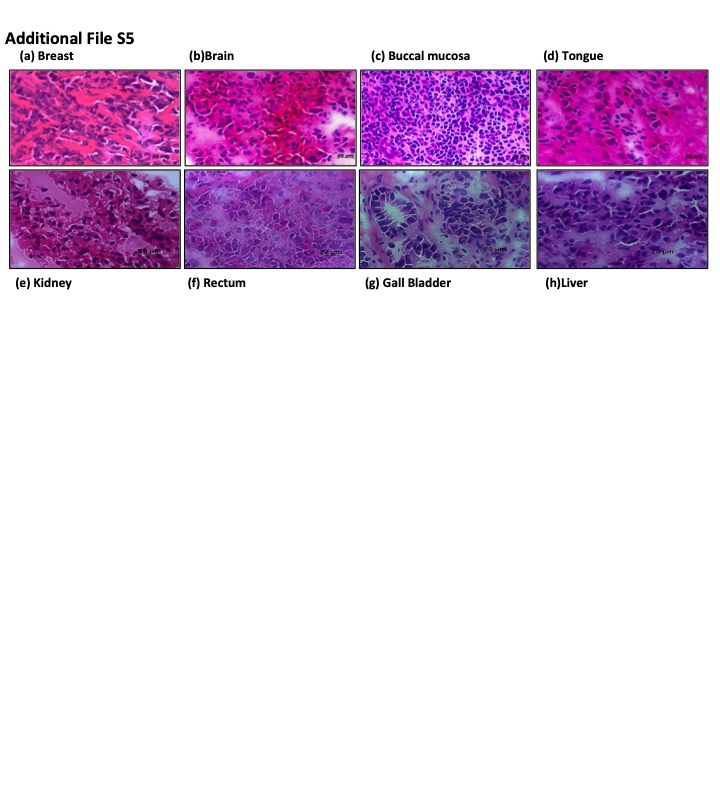

Supplement: Supplementary file 5 — Additional file 5. (a-h) Representative images of Hematoxylin and Eosin staining of human tumor samples, assessed by pathologist for tumor content. Samples were derived from tumors of eight different tissue origins. Magnification – 40X, Scale bar- 20 μm. [file 13148_2019_800_MOESM5_ESM.jpg]
