# Supplementary material for: Elevated HDAC activity and altered histone phospho-acetylation confer acquired radio-resistant phenotype to breast cancer cells
Source: Clin Epigenetics. 2020 Jan 3;12:4. doi: 10.1186/s13148-019-0800-4 (PMC6942324; doi:10.1186/s13148-019-0800-4)
Supplement: Supplementary file 6 — Additional file 6. Details of the antibodies used in the study. [file 13148_2019_800_MOESM6_ESM.docx]

Antibodies used and their dilutions:

| S.No | Protein/PTM | Catalogue no. | Company | Dilution | Purpose |
| --- | --- | --- | --- | --- | --- |
| 1. | Histone H1 | sc-8030 | Santa Cruz | 1:500 | Western Blotting |
| 2. | HP1α | 2616S | CST | 1:2000 and 1:200 | Western Blotting and Immuno-fluorescence |
| 3. | Histone H3 | 05-499 | Millipore | 1:2000 | Western Blotting |
| 4. | H3K27ac | 4729 | Abcam | 1:3000 | Western Blotting |
| 5. | H3K4me3 | 8580 | Abcam | 1:3000 | Western Blotting |
| 6. | H3K9ac | 06-599 | Millipore | 1:1500 | Western Blotting |
| 7. | H3K9me3 | 8898 | Abcam | 1:4000 | Western Blotting |
| 8. | H4K20me3 | 9053 | Abcam | 1:4000 | Western Blotting |
| 9. | H3S28p | 5169 | Abcam | 1:2000 | Western Blotting |
| 10. | H3S10p | 06-570 | Millipore | 1:2000 | Western Blotting |
| 11. | H3S10pK14ac | 07-181 | Millipore | 1:2000 | Western Blotting |
| 12. | H3K56ac | 76309 | Abcam | 1:2000 | Western Blotting |
| 13. | γH2AX | 05-636 | Millipore | 1:5000 | Western Blotting |
| 14. | PP1α | 07-273 | Millipore | 1:4000 | Western Blotting |
| 15. | MKP-1 | SC-370 | Santa Cruz | 1:3000 | Western Blotting |
| 16. | Msk1 | 99412 | Abcam | 1:1000 | Western Blotting |
| 17. | Msk2 | 99411 | Abcam | 1:2000 | Western Blotting |
| 18. | ERK1/2 | SC-93 | Santa Cruz | 1:2000 | Western Blotting |
| 19. | pERK1/2 | 4370p | CST | 1:2000 | Western Blotting |
| 20. | P38 | SC-728 | Santa Cruz | 1:2000 | Western Blotting |
| 21. | pP38 | 4511p | CST | 1:2000 | Western Blotting |
| 22. | Beta actin | A-5316 | Sigma | 1:10000 | Western Blotting |
| 23. | α-tubulin | ab-4074 | Abcam | 1:200 | Immuno-fluorescence |
| 24. | PKH reagent | PKH26 | Sigma | 4μM from 10mM stock | Cell membrane visualization |
