# Supplementary material for: Elevated HDAC activity and altered histone phospho-acetylation confer acquired radio-resistant phenotype to breast cancer cells
Source: Clin Epigenetics. 2020 Jan 3;12:4. doi: 10.1186/s13148-019-0800-4 (PMC6942324; doi:10.1186/s13148-019-0800-4)
Supplement: Supplementary file 7 — Additional file 7. Sequence of primers used in the study. [file 13148_2019_800_MOESM7_ESM.docx]

**Additional file 7 - Sequences of the primers used in the manuscript for real-time PCR.**

| **S.No.** | **Gene** | **Forward Primer (5`-3`)** | **Reverse Primer (5`-3`)** |
| --- | --- | --- | --- |
| 1. | KLF4 | CCCACCTTCTTCACCCCTAGA | CTTCCCCTCTTTGGCTTGGG |
| 2. | LIN28A | GGAGGCACAGAATTGAGCCA | CAGTGCCAACTAGCCCCAAT |
| 3. | MYC | TGCCCATTTGGGGACACTTC | TGCTGGTTTTCCACTACCCG |
| 4. | NANOG | TAATAACCTTGGCTGCCGTCT | AAAGCCTCCCAATCCCAAACA |
| 5. | SOX2 | TTCATCGACGAGGCTAAGCG | AACTGTCCATGCGCTGGTT |
| 6. | RPS13 | GCTCTCCTTTCGTTGCCTGA | ACTTCAACCAAGTGGGGACG |
| 7. | BRCA1 | TTCGTATTCTGAGAGGCTGCTG | GTAATTCCCGCGCTTTTCCG |
| 8. | ATR | TTTTGGCCTCCACACGGC | GCACTAGTCAACCACGCCAA |
| 9. | ATM | CTAAGTCGCTGGCCATTGGT | TCTGGAGGAAGAAGCAACGC |
| 10. | P53 | TAACAGTTCCTGCATGGGCG | TGGTGAGGCTCCCCTTTCTT |
| 11. | HDAC1 | ATATCGTCTTGGCCATCCTG | TGAAGCAACCTAACCGATCC |
| 12. | HDAC2 | GGGAATACTTTCCTGGCACA | ACGGATTGTGTAGCCACCTC |
| 13. | HDAC3 | TGGCATTGACCCATAGCCTG | GCATATTGGTGGGGCTGACT |
| 14. | HDAC4 | TCGCTACTGGTACGGGAAAAC | AGAGGGAAGTCATCTTTGGCG |
| 15. | HDAC5 | ACTGTTCTCAGATGCCCAGC | TGGTGAAGAGGTGCTTGACG |
| 16. | HDAC6 | AGTGGCCGCATTATCCTTATCC | ATCTGCGATGGACTTGGATGG |
| 17. | HDAC7 | TTCCTGAGTGCAGGGGTAGT | CATCGCCAGGAGGTTGATGT |
| 18. | HDAC8 | ATAACCTTGCCAACACGGCT | CTTGGCGTGATTTCCAGCAC |
| 19. | HDAC9 | ACTGAAGCAACCAGGCAGTC | TTCACAGCCCCAACTTGTCC |
| 20. | HDAC  10 | CTGGCCTTTGAGGGGCAAAT | CAGCAGCGTCTGTACTGTCA |
| 21. | HDAC  11 | CCGGAAAATGGGGCAAAGTG | TAAGATAGCGCCTCGTGTGC |
